# Editorial: Cellular neuropathology of hearing loss

**DOI:** 10.3389/fncel.2026.1877741

**Published:** 2026-05-25

**Authors:** Soledad Levano, Barbara Jane Morley, Marisa Zallocchi

**Affiliations:** 1 University Basel Hospital, Basel, Switzerland; 2 Boys Town National Research Hospital, Omaha, NE, United States; 3 Biomedical Sciences Department, Creighton University, Omaha, NE, United States

**Keywords:** afferent ribbon synapse, auditory neuropathology, central auditory, cochlea, diabetic hearing loss, hearing loss, HIV, ototoxic model

Hearing loss is multifactorial in nature, and its causes include both genetic and lifestyle-related factors. Mechanistic studies have shown that environmental stressors—particularly noise exposure and ototoxic substances—trigger cascades of oxidative stress, glutamate-induced excitotoxicity, and apoptosis, which ultimately lead to the loss of hair cells and synaptic connections—processes known as cochlear synaptopathy. While this proposed model was fundamental, it largely viewed hearing loss as a peripheral and cell-autonomous disorder of the cochlea, with only limited consideration given to systemic or regulatory influences.

The aim of our Research Topic was to investigate the complexities of the cellular mechanisms contributing to hearing loss and to identify the pathophysiological processes that impair hearing function. In this context, research on aging has expanded our understanding of the temporal patterns of degeneration. The study by Dörje et al. confirmed the early vulnerability of afferent synapses (Kujawa and Liberman, 2009) and demonstrated that degeneration of efferent system occurs later (Altschuler et al., 1984). This study is significant because it complements earlier work by demonstrating that age-related hearing loss does not occur uniformly across all cochlear circuits and by providing precise temporal and spatial mapping. The study points to a temporal and region-specific degeneration pattern, in which afferent ribbon synapses undergo an earlier and more pronounced decline than efferent ones. This leads collectively to reduced stability of cochlear amplification and impaired homeostasis.

The expanded cellular perspective is further refined by mechanistic insights from ototoxic injury models. Manickam and Zallocchi addressed the critical issue of the limited validity and standardization of existing animal models. Furthermore, the study highlights the persistent discrepancy between preclinical and clinical contexts. While patients typically receive repeated, cumulative doses of cisplatin, many animal studies rely on acute, high-dose regimes that do not accurately replicate the temporal dynamics or systemic effects of chemotherapy. Notably, the authors introduced a mythological innovation to improve reproducibility and align protocols toward translational research.

The transition toward mechanistic complexity is particularly evident in Kurabi et al.'s study. This study demonstrated that protecting the cochlea requires balanced modulation

of multiple interacting pathways. Since it is known that traditional “one-target-one-drug” approaches often fail in cases of multifactorial etiologies, the concept of multi-target therapy has gained importance in drug design (Ramsay et al., 2018). Kurabi et al. provided direct evidence in the cochlea by using subthreshold doses to activate six different signaling pathways. The authors introduced a new perspective, according to which the effective path to protecting the cochlea does not lie in the maximal activation of signaling pathways and an excessive combination of signaling pathways, but rather in optimizing the balance at the system level. Furthermore, the combination of therapeutics exhibits non-linear responses and should therefore be applied within an optimal range, as both insufficient and excessive interventions can reduce efficacy and exacerbate damage. This supports a model in which hearing loss arises from dysregulation of interconnected cellular networks, rather than a single dominant signaling pathway.

Consistent with the Research Topic's aim to explore diverse cellular mechanisms, recent studies have expanded the understanding of auditory neuropathology beyond systemic disease contexts. In relation to diabetes, Bian et al. have developed a biomarker-based predictive model for diabetic hearing impairment. This nomogram prediction model integrates circulating biomarkers such as non-coding RNAs and metabolic markers. By combining markers reflecting cumulative blood glucose levels, oxidative stress, inflammation, and apoptosis, the authors moved away from single-biomarker approaches toward multifaceted systems, reflecting broader trends in personalized medicine. While a technology enabling the detection of multiple markers of kidney damage has already been reported for diabetes (Li et al., 2024), the authors addressed the lack of accessible, non-invasive tools for the early detection of hearing disorders in systemic diseases. This work establishes a link between metabolic and epigenetic dysregulation as a risk factor for cochlear vulnerability laying the groundwork for integrating non-invasive diagnostics for hearing impairment into routine clinical assessment.

Similarly, Woods et al. demonstrated that systemic viral infection impairs central auditory processing, with reduced cortical activation suggesting a dysfunction beyond the cochlea. In contrast to earlier studies on HIV in children, which demonstrated cognitive impairments (Boivin et al., 2018), and abnormalities in auditory brainstem (Matas et al., 2015), the findings of this study showed that HIV-infected children exhibit reduced activation in the auditory cortex despite presumably intact peripheral input. This suggests a dysfunction at the level of primary sensory cortical coding. This expands the definition of pediatric HIV to include the involvement of central neural circuits.

The review by Förster et al. emphasizes that, at the interface between these systems, hypertension and noise-induced hearing loss are not merely separate conditions but are often linked by shared oxidative, vascular, and inflammatory signaling pathways. The authors highlight how vascular dysfunction and reduced cochlear perfusion interact with noise-induced metabolic stress, leading to synergistic cochlear injury. The interaction model between noise-induced hearing loss and hypertension underscores the role of systemic physiological states in modulating cellular vulnerability. The authors also highlight innovative medical treatment methods.

Taken together, these six studies describe advances in the molecular and cellular mechanisms of hearing loss and classify it as a multiscale cellular neuropathology, characterized by time-dependent synaptic degeneration, hair cell vulnerability, efferent dysregulation, vascular impairments, and central auditory dysfunction. This also underscore the trend toward clinically translatable models of hearing loss, emphasizing multi-level biomarkers, reproducible experimental conditions, and combinatorial therapeutic strategies. Such approaches are essential to address the complexity of auditory system biology and to advance toward precision medicine in hearing disorders.
